# Screening and Scale-Up of Nanofiltration Membranes for Concentration of Lactose and Real Whey Permeate

**DOI:** 10.3390/membranes13020173

**Published:** 2023-01-31

**Authors:** Katrin Hofmann, Christof Hamel

**Affiliations:** 1 Applied Biosciences and Process Engineering, Anhalt University of Applied Sciences, Bernburger Straße 55, 06366 Koethen, Germany; 2 Institute of Process Engineering, Otto von Guericke University, Universitaetsplatz 2, 39109 Magdeburg, Germany

**Keywords:** polymeric membrane, nanofiltration (NF), concentration of lactose and whey permeate, process parameters, flat-sheet module, scale-up, spiral wound module, molecular weight cut-off (MWCO)

## Abstract

In dairy industry huge quantities of whey accumulate as a by-product. In particular the containing lactose was not produced profitably in the past. Thus, the trend goes towards modification and sustainable use of lactose for which a concentration step is required. Nanofiltration (NF) has shown to be a good choice since partial demineralization can be realized in parallel. Therefore, in this study, 10 commercial polymer NF membranes were studied in detail and systematically for their suitability to concentrate lactose, with the proviso of high flux and high to complete rejection. Preliminary trials were conducted with flat-sheet membranes and a lactose model solution and the influence of transmembrane pressure (TMP), temperature and lactose concentration was studied. Finally, results were evaluated by using spiral wound modules and real industrial whey permeate. The results offered that a membrane screening is essentially since no correlation between molecular weight cut-off (MWCO) and permeate flow could be found. The conclusions found for the lactose model solution were in good agreement with the whey permeate, but as the ions contribute to the osmotic pressure of the feed the deviations increase in the course of concentration since ions are also partly retained.

## 1. Introduction

The utilization of whey has undergone major changes in recent years and decades. In order to produce 1 kg of cheese, around 10 L of milk are used and around 9 L of whey result as a by-product [1]. In the past whey was disposed of as waste in sewage and rivers, which led not only to environmental problems because of a high chemical oxygen demand (COD) between 50–60 kg/m³ and high biological oxygen demand of around 30–40 kg/m³ but also to a loss of valuable resources [1,2,3]. Besides legal regulations regarding waste water treatment and grown scientific knowledge of the value of whey components, the interest and awareness has raised with respect to increasing sustainability that are characterized by value chains with complete utilization of all milk components along with recycling product wastewater treatment [4,5]. In particular, the lactose as a main component of the side-product whey has not been used profitably in the past since the production has not been economically beneficial and the traditional lactose market is rather stagnating [4]. Additionally, around the world there is a great diversity in the distribution of lactose intolerance among adults ranking between 1% in the Netherlands and 98% in Southeast Asia [6]. Nevertheless, globally the dairy market is still growing because demand for dairy products in developing countries is growing along with income [7]. Therefore, the dairy industry is looking for alternative ways of lactose utilization. There are many approaches reported in literature, such as the synthesis of the prebiotics galacto-oligosaccharides, lactulose, lactitol, lactobionic acid, biogas or bioethanol [4,8,9] that require concentrated lactose to enhance reaction rates and synthesis efficiency, respectively [8,10]. Since the structure of dairy industry around the world is diverse, a mixture of many small family dairies and a few large industrial dairies [7], it is in general reasonable to concentrate the lactose prior to further processing or for cost-effective transport. In recent years nanofiltration (NF) as a powerful tool for concentration of lactose has gained raising interest because in parallel a partial demineralization can be realized that is often essential for subsequent processes [11,12]. Conventional demineralization strategies of whey include ion exchange and electrodialysis which are accompanied by high investment and operating costs [13]. In contrast, NF is, like reverse osmosis (RO) and ultrafiltration (UF), a pressure-driven membrane process and lies in between these two filtration processes in terms of operating pressure and separation limits and thus has corresponding characteristics of both RO and UF [14].

Previous studies have shown that NF is generally suitable for concentrating lactose (molecular weight = 342 g/mol) in whey permeate, with the positive side effect of partial demineralization compared to conventional evaporation, where all compounds within the feed are concentrated [3,11,12,15]. The focus of these studies was primarily on checking the feasibility of the process and investigating the influence of selected operating parameters whereas classification and comparability of the results is difficult because different membranes and/or operating parameters were used that influence the extent of demineralization. The impact of the choice of membrane was demonstrated by Räsänen et al. who compared four commercial NF membranes that varied in their solute retentions [12]. With rising volume concentration ratio (VCR) in the course of concentration the retention of ions slightly decreases and the degree of demineralization is increased [11,15]. The demineralization can be further enhanced through diafiltration (DF), which is accompanied by a greater loss of lactose [11]. Thus, DF is associated with a trade-off between the degree of demineralization and loss of lactose, whereas a mathematical model can support the optimization of the DF process as described in [16].

Therefore, the focus of this study is the comparison of ten commercially available membranes within the same experimental setup and reproducible experimental conditions. The aim is to identify the membrane with the best overall performance in terms of flux and retention in the sense of an ecological and efficient process and to get a better understanding of the extent of influence of different operating parameters.

Generally, it would be favorable to be able to model the membrane process in order to reduce the number of trials and thus the costs for the process design [17]. The experiments performed and the data derived provide a basis for that. However, the filtration process is based on complex interactions that determine the outcome of the overall process. The main influencing factors include the membrane properties, the composition of the feed and the operating parameters, which also interact with each other and also may affect significantly the three thermodynamic driving forces: concentration, pressure and voltage differences [18,19]. Nowadays the precise membrane composition is mostly a trade secret, since the membrane manufacturers are very secretive about the exact composition and structure of the membranes [20,21]. This in turn makes predictions even more difficult, especially in the context of interactions between membrane and complex feeds. Also, because structural properties of the pores and the solute, like size, shape and length and physicochemical properties like zeta potential and hydrophilicity influence the permeation behavior [22]. The separation mechanism in NF is based on charge and size exclusion [22,23]. Flow and selectivity of neutral solutions in NF can be described reasonably well through the solution-diffusion model originally formulated for RO. In contrast, the complexity of ionic solutions is very pronounced, since interactions with the membrane as well as with each other take place depending among other things on pH, temperature and concentration [14,24,25]. Various research approaches were pursued with single salts as well as with salt mixtures under different process conditions like pH and temperature in order to gain a better understanding of the interactions and extent of influence [14,23,26,27,28]. However, due to the complexity of the interactions involved in the nanofiltration of electrolyte solutions, the membrane performance cannot be fully predicted and further research is needed, especially as complex mixtures are a great challenge [14,17,28]. Actually, real industrial whey is a rather complex mixture of compounds, many of which may interfere with the membrane surface, or compounds that could interact with each other in different ways. A further challenge is that there is not just one type of whey but that the composition is diverse depending on seasonal variation within the raw milk but also due to different manufacturing, production and processing processes [5,13]. Thus, preliminary trials are still essentially for an overall economically and ecologically process design, ideally with the desired process parameters and feed [14,17]. Therefore, in terms of systematics, first orienting trials were carried out with a model solution consisting of pure lactose to initially exclude the complex interactions caused by the whey permeate matrix with its ions, acids and other components like amino acids, peptides and proteins. Finally, these data were compared with the performance when industrial whey permeate had been used.

In summary, this study has three main objectives:

1. Membrane screening—Out of 10 membranes, identification of those membranes with the best performance in terms of high flux and high retention of lactose.
2. Determination of process parameter settings and its extent of influence for achieving high filtration fluxes and high retention of lactose.
3. Scale-up and evaluation of the influence of industrial whey permeate on the course of filtration compared to the lactose model system.

## 2. Materials and Methods

### 2.1. Feedstock

All feed solutions were preserved from microbiological spoilage by addition of 0.02% (*w*/*w*) sodium azide (Carl Roth, Karlsruhe, Germany) in order to avoid the formation of biofilms that could affect the results. For preliminary experiments a model solution was used that was prepared by dissolving a pharmaceutical-grade α-lactose monohydrate (Carl Roth, Karlsruhe, Germany) in deionized water in the amounts needed to reach the desired lactose concentrations of approximately 25, 50 and 75 g/L.

Fresh sweet whey permeate was taken from a feed line of an industrial filtration process of a local dairy plant where it had been separated by a 10 kDa UF membrane and was stored at 4 °C until use. The average composition of the sweet whey permeate used is shown in Table 1.

### 2.2. General Experimental Setup of Nanofiltration Trials

**Figure 1:**
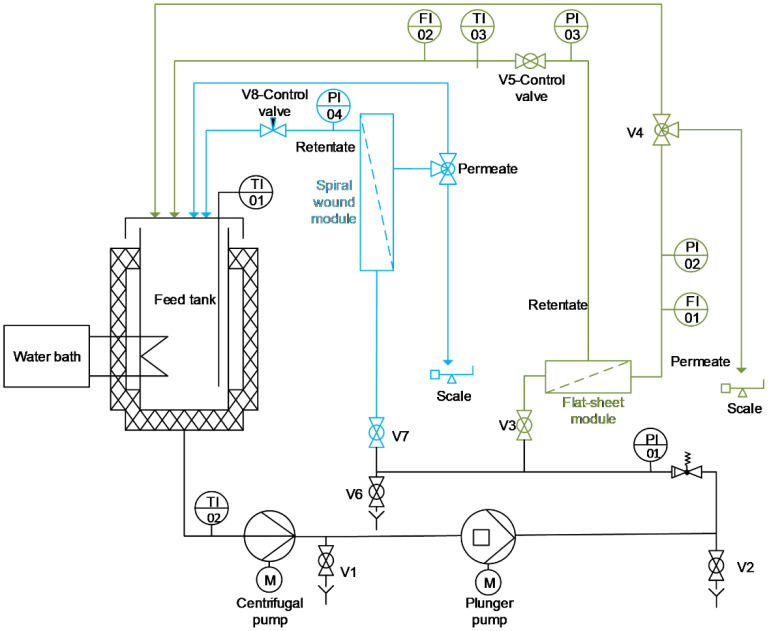
Piping and instrumentation diagram of filtration plant (green—flat-sheet module circuit; blue—1812’’ spiral wound module circuit).

The general structure and/or flow sheet of the semi-pilot scale filtration plant used for the experiments was provided by inocermic (Hermsdorf, Germany) is shown in Figure 1.

**Figure 2:**
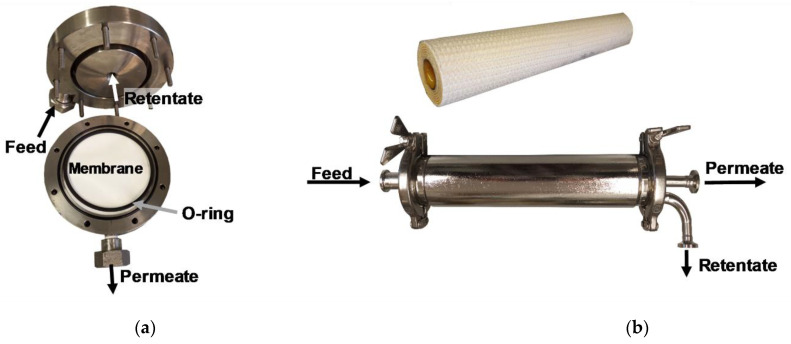
Visualization of modules used. (**a**) flat-sheet module; (**b**) spiral wound membrane and housing (1812″).

The plant was designed for tests in crossflow mode and was equipped with both a pressure housing for flat-sheet membranes and one for 1812″ spiral wound modules realizing scale-up. Figure 2 illustrates the construction of the flat-sheet test cell. The feed flew parallel to the membrane and the retentate was recirculated perpendicularly to the membrane surface.

All pipes and the feed tank were insulated and temperature was kept constant during operation using a water bath (F32, Julabo, Seelbach, Germany) that was connected to the double jacket of the feed tank. The actual temperature in the feed tank was measured directly by the digital temperature sensor TI-01 in the feed using a thermocouple. The maximum capacity of the feed tank was 12 L.

The plant had a centrifugal pump (CDX/A 70/05, EBARA Pumps Europe, Gambellara, Italy) that acted as a feed pump for the high pressure plunger pump (271 D1110, Cat Pumps, Idstein, Germany) which was responsible for the flow and pressure generation during the process. Both pumps were controlled via frequency converters in order to set the desired volume flow. For the flat-sheet trials a volume flow of 0.30 m³/h had been used while with the spiral wound module a volume flow of 0.43 m³/h had been set. The retentate-side pressures were adjusted manually to the desired value by the control valves. In case of the flat-sheet module the control valve V5 was used and the pressure was measured by the pressure gauge PI-03. For the spiral wound module the transmembrane pressure (TMP) was adjusted by the needle valve V8 and monitored by the pressure gauge PI-04.

The permeate weight for calculating the permeate flux was monitored by a scale (Scout STX6201, OHAUS Europe, Greifensee, Switzerland).

### 2.3. Filtration Test Scheme

#### 2.3.1. Membranes and Experimental Matrix

In this study 10 commercially available NF membranes from 3 different manufacturers were investigated. Table 2 summarizes the membranes used within this study and gives an overview of their properties in terms of specified molecular weight cut-off (MWCO), maximum operating pressure and temperature as well as membrane material provided by the manufacturers.

The experimental matrix had 3 stages and is summarized in Table 3. All trials were carried out at least in duplicate. At the beginning of every trial the actual membrane and its performance was checked and characterized via pure water flux measurements at different TMP steps from 0.5 MPa up to maximum 3.5 MPa in 0.5 MPa steps at the temperature of the respective trial. All water used in the filtration experiments was deionized water with 0.02% (*w*/*w*) sodium azide. The water flux measurements were also used to evaluate the cleaning efficiency.

The first stage started with a TMP-screening of all 10 membranes in order to be able to compare the performance of the membranes in terms of retention and permeate flux. Based on these results, in the second step (parameter studies) a pre-selection of 5 membranes was carried out, in which the influence of various process parameters was examined in more detail.

These first 2 test stages were carried out with flat-sheet membranes that were delivered as dry sheets and cut to a circular diameter of 9.1 cm to fit the test cell. After subtraction of the O-ring area, this results in an effective filtration area of 0.0053 m². At the last stage (scale-up), experiments were carried out with 1812” spiral wound modules, concrete with the NFS from Synder Filtration (Vacaville, CA, USA) with a filtration area of 0.28 m² and the XN45 from MANN+HUMMEL (Ludwigsburg, Germany) with a filtration area of 0.20 m². Both spiral wound modules had a feed spacer thickness of 46 mil.

#### 2.3.2. First Stage—TMP-Screening

Prior to every experiment the water flux was measured as explained in Section 2.3.1. The TMP-screening was conducted at 20 °C with 5 kg of lactose model solution with a lactose concentration around 25 g/L which was based on the initial lactose concentration of the industrial sweet whey permeate. In order to maintain a constant composition of the feed, the permeate was regularly returned to the feed tank which means the experiments were done in recirculation mode. The TMP was increased in steps of 0.5 MPa, starting with 0.5 MPa to maximum of 3.5 MPa or the maximum operating pressure of the membrane followed by a stepwise decrease of the TMP to prove hysteresis. At every investigated TMP after at least 10 min at this TMP or when the permeate flux was stable, the permeate flux was measured and a sample of the feed and the permeate was taken for lactose quantification via HPLC (Section 2.4.1) in order to calculate the lactose retention.

After each experiment the plant and the membrane was firstly thoroughly flushed pressureless with tap water at 45 °C followed by deionized water at room temperature. In order to profoundly clean the flat-sheet test cell, it was disassembled and the membrane was carefully rinsed with deionized water and stored in deionized water with 0.02% sodium azide inside the reassembled test cell. Water flux measurements confirmed the successful cleaning procedure.

#### 2.3.3. Second Stage—Parameter Studies

With the 5 selected membranes (see Table 3), parameter studies were carried out in concentration modes with 7 kg of lactose model solution, i.e., after 10 min with recirculating permeate, the feed was concentrated to a VCR of 2.25. As a starting point for the comparative evaluation of the influence of various process parameters the following standard process parameters were set: ϑ = 20 °C, TMP = 2 MPa and an initial lactose concentration of 25 g/L. The permeate flux was measured at defined VCR (every 0.25 step) and every 30 min. At these defined VCR there were also samples taken for lactose quantification from the feed, the permeate out of the permeate pipe and from the cumulating permeate. The experiments were conducted at 45 °C to investigate the influence of the temperature. The influence of the TMP was investigated at 3 MPa. The combined effect of temperature and TMP was studied at 45 °C and 3 MPa with those membranes that tolerated the combination of these process parameters. Finally, the impact of increasing initial lactose concentration within the feed was studied in detail. Because of the limiting maximum tank volume, the experiments were divided in separate experiments with an initial lactose concentration of 50 g/L and 75 g/L. Depending on the tolerance limits of the membranes those trials were carried out either at 45 °C and 2 MPa (membranes from Synder Filtration) or at 45 °C and 3 MPa (the other membranes). The cleaning procedure was identical as for the TMP-screening.

#### 2.3.4. Third Stage—Scale-Up

The scale-up was carried out with those 2 membranes that had revealed the best performance at the previous stages. The experiments were conducted at different process variables for each membrane, because of differences in the maximum operating pressure at temperatures above 35 °C. In order to compare the performance of flat-sheet membranes with spiral wound membranes and evaluate the potential of experiments based on flat-sheet membranes, firstly the lactose model solution was concentrated. In case of the NFS from Synder Filtration the concentration has been performed at 45 °C and a TMP of 2 MPa while with the XN45 from MANN+HUMMEL the concentration was carried out at 45 °C and 3 MPa as well as 20 °C and 3 MPa. In a second step the lactose in sweet whey permeate was concentrated with the same process parameters for the NFS and at 20 °C and 3 MPa for the XN45.

The general procedure of the concentration trials was as followed: As it was intended to reach approximately 200 g/L lactose within the concentrate and the initial lactose concentration was given by the amount in the sweet whey permeate, the maximum tank volume was not sufficient for one whole process. Therefore, the trials started with the maximum feed volume of 14.5 L that was recirculated for 10 min and then the concentration was carried out until a VCR of 2.5 was reached. This procedure was repeated 3 times and the resulting concentrate of all 3 runs was united and 14.5 L of this retentate were concentrated to a VCR of 3, resulting in a total VCR of 4.5. At defined VCR (every 0.5 step, except at the beginning additionally at 1.25) the permeate flux was measured and samples were taken for lactose quantification from the feed, the permeate out of the permeate pipe and from the cumulating permeate. When the whey permeate was used as feed, the conductivity and ion composition of the token samples was additionally analyzed. 

The solute rejection (*R_j_*) of the lactose and the ions points to the percentage of solute that does not pass the membrane and was calculated based on the following Equation (1) [29].

(1) $$R_{j}\left(\%\right)=\left(1-\frac{c_{j,p}}{c_{j,f}}\right)\times 100$$

where *c_j,p_* and *c_j,f_* are the solute concentration in the permeate pipe and the feed concentration at a defined VCR. The loss respectively removal of solute was calculated based on balancing the masses of the solute in the feed and in the cumulating permeate as follows:

(2) $$Loss_{i}\left(\%\right)=\frac{m_{i,\;p}}{m_{i,\;f}}\times 100\%$$

where $m_{i,\;p}$ is the mass of the solute in the cumulative permeate and $m_{i,f}$ is the mass of the solute in the initial feed.

When working with the lactose model solution the cleaning procedure was the same as for the TMP-screening. In case whey permeate was used as feed, the cleaning procedure was extended by an additional cleaning step with an enzymatic cleaning agent (Ultrasil 53, Ecolab, Monheim am Rhein, Germany) at a concentration of 1% after the system had been flushed thoroughly with tap water. The enzymatic cleaning was performed pressureless at approximately 40 °C under constant recirculation of retentate and permeate for 30 min. Afterwards the plant was repeatedly flushed with tap water and then the previously described cleaning routine followed.

### 2.4. Analysis Methods

#### 2.4.1. Lactose Quantification

Quantification of lactose concentration before and during the filtration process was realized via HPLC Chromaster^®^ HPLC system (VWR, Darmstadt, Germany). The column Vertex Plus Eurokat Na, 300 × 8 mm ID with a 30 × 8 mm ID precolumn, particle size 10 µm (Knauer GmbH, Berlin, Germany) was used with a flow rate of 0.25 mL/min, ultrapure water with 0.02% sodium azide (*w/v*) as eluent and a sample injection volume of 10 µL. The column was kept at a temperature of 85 °C and the detection was carried out at 40 °C with a refractive index detector.

#### 2.4.2. Quantification of Ions and Conductivity

Ions were analyzed by the ion Chromatograph DX-100 (Dionex Corporation, Sunnyvale, CA, USA), equipped with a conductivity detector. All samples were filtered through a 0.2 µm syringe filter before analysis, if appropriate diluted with deionized water and 25 µL of the sample were injected and measured at room temperature. For anions the column Dionex™ IonPac™ AS14 (4 × 250 mm) with precolumn Dionex™ IonPac™ AG14 (4 × 50 mm) was used with a flow rate of 1 mL/min, 3.5 mM Na_2_CO_3_ and 1 mM NaHCO_3_ as eluent. Cations were analyzed using a Dionex™ IonPac™ CS 12A (4 × 250 mm) column with a Dionex™ IonPac™ CG14 (4 × 50 mm) precolumn, by using a mobile phase of 20 mM methanesulfonic acid, with a flow rate of 1 mL/min.

The conductivity was measured with a WTW conductimeter LF 539 (WTW, Weilheim, Germany) using nonlinear temperature compensation mode based on the reference temperature of 25 °C.

## 3. Results and Discussion

### 3.1. Screening and Preselection of Membranes

Within the scope of this study, NF membranes were to be examined with regard to their suitability for concentrating lactose (molecular weight (MW) = 342 g/mol). When searching for appropriate membranes the MWCO was used as the most important criterion for the selection, but it was found that the specifications in this regard varied greatly. In contrast to UF or microfiltration (MF) membranes, in the majority of cases ranges were given, e.g., for the XN45 300-500 Da and only for the membrane from KOCH Membrane Systems there was one value specified as MWCO. The given number of the MWCO expresses in general the molecular weight of a compound that is rejected to 90% by the given membrane [29,30]. However, one challenge concerning the MWCO is, that there exists no binding definition or methodology and manufacturers may use different methods and feed solutions for determination [30]. Therefore membranes with a higher MWCO than the MW of lactose were also included in the investigations. Additionally this approach was based on the following assumptions: On the one hand, it was supposed that higher MWCO tended to be associated with higher fluxes [3,23], which could result in an overall increase in process efficiency and profitability, although this might entail some losses of lactose. On the other hand, whey permeate contains a large number of compounds and minerals besides lactose [3,5,13]. Therefore, when using membranes with a larger MWCO, the demineralization could possibly also be promoted or potential interfering components that could impair the flux might unfold their effect less strongly. For that reason, as a first step, it was necessary to examine the eligible membranes with regard to their retention capacity for the target compound lactose and the resulting flux. This was realized as an important part of a TMP-screening.

In order to check the reproducibility of the results, tests were carried out with the same membrane as well as with several sections of the same provided membrane lot and, in the case of the NFG, with cuts from different lots. This strategy ensures the testing of a representative sample. In the case of membranes that were used repeatedly and those from the same batch, the curves basically corresponded very well, so that good reproducibility of the tests could be assumed. The data shown represent the mean values of the respective tests.

**Figure 3:**
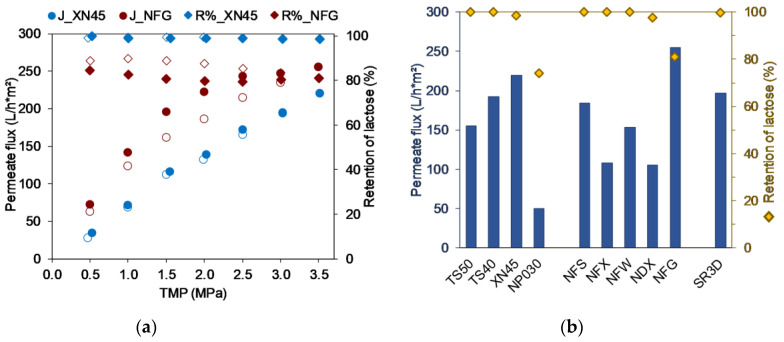
(**a**) Impact of varying transmembrane pressure (TMP) on permeate flux (J) and retention (R%) of lactose (filled icons: stepwise increase of the TMP, empty icons: stepwise reduction of the TMP); (**b**) Comparison of the membrane performance based on permeate flux (bars) and retention of lactose (squares) at transmembrane pressure of 3.5 MPa. Operation parameters in Table 3.

As shown in Figure 3 using the XN45 as an example, all membranes, with the exception of the NFG, showed basically the same course of the curve during the TMP screening, i.e., a stepwise increase of the retentate-side pressure led to a linear increase in flux. This indicates that the permeate flux is a result of the membrane resistance since the process is not mass transfer limited [31]. These observations are in good agreement with the results of [11,15], whereas the studies of [3,15] demonstrate that mainly remaining proteins in whey are responsible for critical flux phenomena during NF, where a further increase in TMP does not result in an equivalent increase in mass flow through the membrane.

However, with the NFG above 2 MPa critical flux phenomena appeared. Since this membrane also offered the highest flux within the TMP-screening compared to the other membranes, it was assumed that the support layer might impede the mass flow, so that a further increase in pressure does not lead to a further linear increase in flux. Chen et al. investigated the effects of different fabrication parameters in the course of the production of a home-made PES NF spiral wound module. Their work demonstrated that with increasing PES-substrate thickness the pure water permeability decreases [21]. An effect through components of the feed solution could be excluded since the results with the other membranes showed that with the investigated lactose concentration no concentration polarization occurred. Furthermore, in the course of the gradual increase and decrease of the TMP a hysteresis effect was found for the NFG and the NP030, i.e., when decreasing the TMP the same flux as before was not reached but the flux was further reduced, as can be seen in Figure 3. In parallel, this led to an increased retention of lactose. Since this behavior only occurred with two of the ten membranes examined, fouling or the formation of a concentration polarization layer due to the feed can be excluded, since otherwise all curves would have to be similar. For the NP030, the compaction as a result of high pressures is known by the manufacturer, i.e., the application of high pressure leads to a compaction of the membrane that is irreversible and thus modifies the membrane performance. This could also be confirmed in repeated experiments with the same membrane. Lower fluxes were always measured in the second run. In contrast, the curves of two consecutive trials with the same NFG membrane were in very good agreement. Therefore, it was assumed that generally compaction does occur, since it shows comparable curves to NP030, but that this is reversible, and might be due to more elastic membrane material components.

Due to the agreement of the curves of the flux as a function of the TMP, it was possible to compare all membranes at one specific TMP in order to identify those membranes with the highest fluxes. In Figure 3 all ten membranes were compared by plotting the permeate flux and the rejection at 3.5 MPa.

Based on the MWCO specified by the manufacturers, there was an expectation with regard to the retention, i.e., that the retention would deteriorate with increasing MWCO, particularly if the MWCO was greater than the MW of lactose with 342 g/mol. Additionally, it was assumed that a higher MWCO would tend to be associated with a higher flux, since the mass flow would be less impeded by increasingly larger pores [3,23]. As a result, the following theoretical order of the membranes was derived from the MWCO specified by the manufacturers: (1)-NFS (175 Da) < (2)-SR3D (200 Da) < (3)-NFX (225 Da) < (4)-TS40 & TS50 (250 Da) <(6)-NFW & XN45 (400 Da) < (8)-NP030 (550 Da) < (9)-NDX (600 Da) < (10)-NFG (700 Da). In contrast, a significantly different order was found when the maximum flux was used for arrangement: (10)-NFG (700 Da) > (6)-XN45 (400 Da) > (2)-SR3D (200 Da) > (4)-TS40 (250 Da) > (1)-NFS (175 Da) > (4)-TS50 (250 Da) > (6)-NFW (400 Da) > (3)-NFX (225 Da) > (9)-NDX (600 Da) > (8)-NP030 (550 Da).

These results elucidate that it is not possible to derive performance with regard to flux based on the given MWCO and that the MWCO is at best indicative. In addition, the membrane manufacturers provide only little information about the membrane material and the exact structure of the support layer [20], so that often only general information are available (Table 2) and therefore these information cannot be used in detail for membrane selection either. This underlines the need for specific, system dependent membrane screening in order to identify the most suitable membrane with the highest flux for the specific filtration task.

The measured retentions were generally in agreement with the expectations, i.e., a MWCO < MW of lactose was associated with a complete retention, while a MWCO > MW of lactose resulted in a partial permeation of lactose. However, the extent of the lactose loss was different in comparable MWCO ranges. For example, in average the NDX (500–700 Da) rejected 97.4% of the lactose compared to the NP030 (500–600 Da) with only 73.7% lactose retention. These results also point out the necessity of a membrane screening, since membrane manufacturers use different methods and substances to determine the MWCO [30]. Thus, it might occur that a membrane with a supposedly not fully suitable MWCO specified by the manufacturer can still have a very good retention and possibly even convince in terms of overall performance (high flux + high retention) for a specific examined separation process near the given MWCO. Finally, the process design depends on various factors, and in particular significantly on the total process time which is affected by the flux. Therefore, those five membranes with the highest fluxes were selected for further investigations, even if complete retention was not achieved with all of these candidates.

### 3.2. Effect of Process Parameters

In the second part of this study the objective was to examine the influence of different process parameters on permeate flux and lactose retention, namely temperature, TMP, the combination of temperature and TMP and increasing lactose concentrations in the feed. Within this context it was particularly important to obey the allowable operating limits, which meant that the combined effect of increased temperature and TMP could not be examined with the membranes from Synder Filtration as these were not as pressure-stable as necessary (3 MPa) at 45 °C.

**Figure 4:**
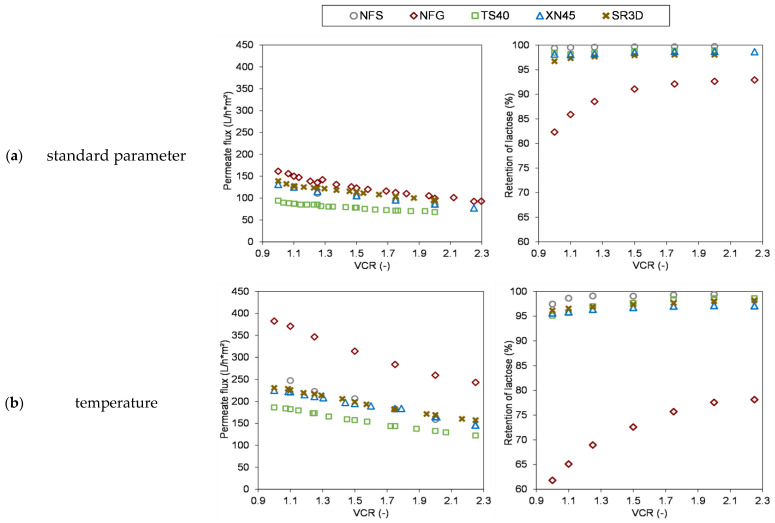

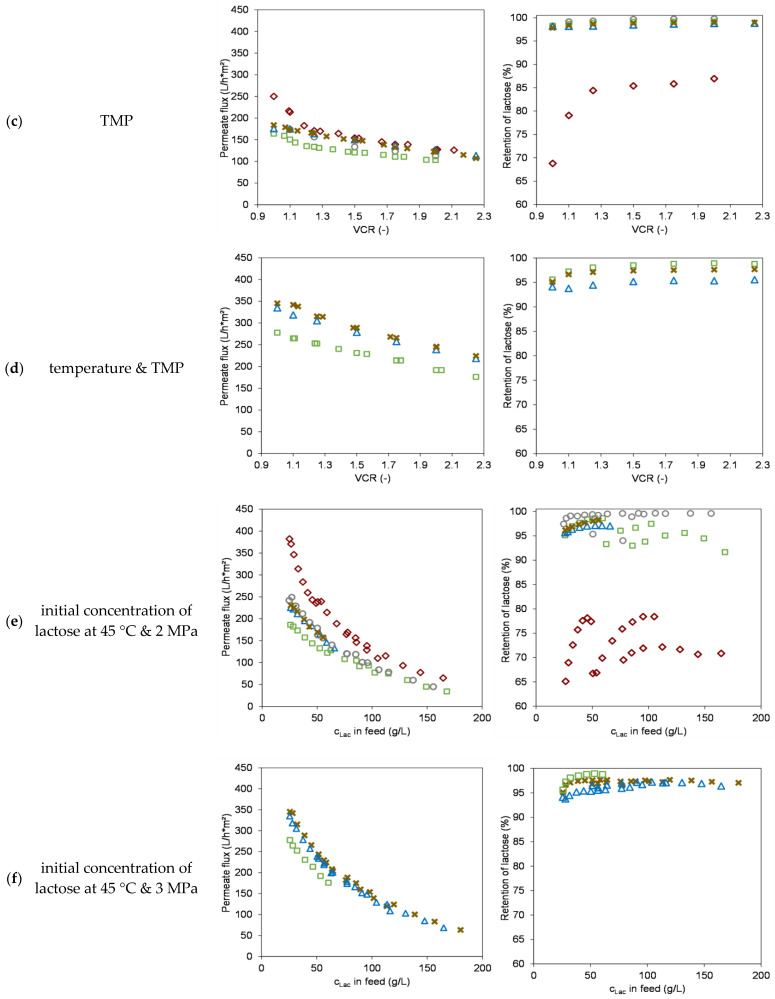
Course of permeate flux (left side) and retention of lactose (right side) during concentration of lactose in dependency of varying process parameters; (**a**) standard parameter (20 °C; 2 MPa; 25 g/L lactose); (**b**) temperature (45 °C); (**c**) transmembrane pressure (TMP) (3 MPa); (**d**) temperature and TMP (45 °C & 3 MPa); (**e**) initial concentration of lactose in feed (45 °C; 2 MPa; 25–75 g/L initial feed-c); (**f**) initial concentration of lactose in feed (45 °C; 3 MPa; 25–75 g/L initial feed-c). Operation parameters summarized in Table 3.

The results are summarized in Figure 4. The influence of the studied parameters was compared with the curves resulting from the concentration of 25 g/L lactose at 20 °C and 2 MPa using those data sets as standard parameters. The NFS, SR3D and XN45 showed very similar flux values in the course of concentration. Only the flux of the TS40 was lower and the NFG showed in agreement with the TMP-screening the highest fluxes. For the NFG this was accompanied by a reduced lactose retention, which improved to about 92% in the course of concentration. The retention of the other four membranes remained at a consistently high level of ≥97%.

Overall, it can be stated that with the exception of the NFG, the magnitude to which the membrane performance responds to a change in a process parameter was very similar for all other four membranes considered.

In several studies the effects of temperature on permeate flux and retention of solutes were investigated. Temperature can influence the membrane morphology and the properties of the feed with its solutes which in turn determine the membrane performance [31,32]. In general, an increase in temperature reduces the viscosity of the feed and thus results in an increased permeate flux [3,15,22,23,32]. While for UF membranes these observations can just be explained by the Hagen-Poiseuille law and show a linear relationship [3], for NF membranes with much smaller pores the underlying mechanisms are much more complex, affecting water permeability and rejection of solutes [22,32]. Tsuru et al. [22] have studied these phenomena extensively. They have carried out the investigations with inorganic ceramic membranes in order to exclude temperature effects on the membrane morphology since it is assumed that ceramic materials do not change when exposed to a temperature increase compared to polymeric materials. As a result they have proposed three potential explanations induced by increased temperatures: (a) The mass transport of water molecules through the micropores may be provoke an activated process promoted through the increase of thermal energy of the water molecules. (b) An increase of the effective pore diameter, because besides the given pore diameter the effective pore diameter is determined by the amount and layer thickness of adsorbed water molecules on the pore walls and this layer decreases with temperature rise. (c) The influence of temperature on the viscosity differs between the inside of the micropores and the bulk solution so that the viscous effect is more pronounced inside the pores [22]. Within this study a similar factor was found for all examined membranes. Increasing the temperature from 20 °C to 45 °C led to a doubling of the permeate flux for the NFS, NFG and TS40. In contrast, the flux of the SR3D increased only 1.7-fold and for the XN45 1.8-fold. The differences between the membrane performances with regard to the retention of the lactose were much more distinct. Precisely, for the NFG with the highest MWCO, an increase in temperature was associated with a decrease in rejection of 15–20%. However, the XN45, whose specified MWCO lay also above the MW of lactose, showed only a slight reduction in lactose retention of about 1.8%. For the other three membranes, an increase in temperature had no effect on the rejection. These differences between the membranes can only be explained by temperature effects on the polymer structure of the membrane material. Generally, both pore-widening and pore-narrowing effects may occur. A temperature increase might enhance the mobility of the membrane polymer chains which in turn might result in an additional polymer relaxation [27]. This might affect the effective membrane thickness and effective pore diameter, which in turn might influence the retention of the components [32]. Yao et al. [31] have determined the MWCO of four polymeric membranes at 20 and 50 °C with a solution of polyethylene glycols of different molar masses. At elevated temperatures they measured higher MWCO and concluded that the temperature rise induces changes in the polymeric membrane structure to the same extent for the open and tight membranes. However, temperature cycling experiments (T = 20 °C→T = 50 °C→T = 20 °C) revealed that these changes were only completely reversible for the tight membranes whereas the membrane performance of the more open membranes was altered due to permanent reorientation of the polymer structure [31]. The water flux measurements before and after the trials of the tested membranes within this study showed comparable curves indicating that no permanent changes in the membrane morphology due to temperature occurred. Several studies reported a rejection drop at elevated temperatures [3,33,34]. Precisely, for uncharged solutes like lactose and fructose an increased temperature was accompanied by a decrease in retention of those solutes. This was explained by the reduced viscosity and thus increased diffusivity due to higher temperatures. As a result, the permeation of neutral solutes was amplified [3,33,34]. Furthermore, changes in the membrane structure have to be considered. Ben Amar et al. [32] investigated the retention of four neutral solutes with MW between 92 and 342 g/mol by a polymeric NF membrane at elevated temperatures. Generally, for all four solutes the rejection decreased as the temperature arose. However, the strongest rejection drop occurred for the intermediate solutes whereas the temperature increase had only a minor impact on the sucrose with a MW of 342 g/mol [32]. These observations might among others explain the differences between the investigated membranes. The decrease in retention was most pronounced for the NFG with the highest MWCO, followed by the XN45 whose MWCO ranges partly above the MW of lactose. It can be deduced from this that it is important to conduct trials at elevated temperatures since the manufacturer’s data on MWCO are usually gained at room temperature and thus the values and as a result the membrane performance at higher temperature might differ [31].

As already observed in the TMP-screening, an increase in TMP leads to a higher permeate flow rate, although the magnitude is less pronounced than the influence of temperature. In general, a pressure increase will at last lead to a limiting pressure where a further increase will not enhance the permeate flux any further. This is among others explained by the osmotic pressure model since the application of higher pressure leads to a further increase of the concentration of the solute near the membrane that is higher compared to the bulk concentration. As a result the increased concentration at the membrane surface reduces the driving force (pressure difference) and thus limits the permeate flux [35]. The TS40 responds most strongly to an increase in TMP (1.6-fold), the effect is least pronounced with the NFG. This supports the hypothesis formulated during the TMP-screening, that the membrane structure of the NFG might impede mass flow. For the NFG the increased TMP also has an adverse effect on retention. The TMP increase from 2 to 3 MPa results in an initial reduction in retention of approximately 13%, with a final difference of 7% at the VCR of 2.25. For the other four membranes, retention remains at a consistent high level.

As the three membranes SR3D, TS40 and XN45 withstand the combination of increased temperature and increased TMP, the combined effect of both parameters could be investigated. It was shown that the combination results in the greatest influence on the permeate flux as expected. With the TS40, the flux triples without changes in retention. For the XN45, the temperature and TMP increase results in a 2.7-fold increase in flux and only a slight decrease in retention of approximately 3.6%. The rejection of the SR3D is unchanged and the flux increased by a factor of 2.6.

In the last part of the parameter study, lactose was concentrated in three stages from 25 g/L to approximately 170 g/L. These experiments were carried out based on the results of the previous trials, i.e., since the temperature within the examined range had the greatest flux-improving effect without any significant reduction in retention, the process was realized at 45 °C (Table 3). Additionally, the solubility of lactose at 20 °C is rather low (15.9 wt%) compared to other sugars which could impede the concentration process. With rising temperatures there is a strong increase in solubility so that from this point of view it is favorable to work at as high temperatures as possible [36]. Since the membranes from Synder Filtration (NFS; NFG) do not have such a high pressure stability at high temperatures, the lactose was concentrated at 2 MPa while with the other three membranes at 3 MPa. The results are summarized in (e) and (f) of Figure 4. Generally, the curves of all membranes are similar. As expected, in the course of concentration the flux continuously decreases due to an increase in osmotic pressure, as described by several authors [11,12,15]. Di Giacomo et al. [36] have shown that the osmotic pressure is not much influenced by the temperature but strongly depended upon the concentration. Thus, the osmotic pressure determines the maximum possible concentration that can be reached with NF as the driving force of pressure difference is finally equalized by the osmotic pressure of the feed. In addition, it can be seen that the curves of the three sub-trials fit very well together and draw a plausible overall curve of the permeate flux in dependency of increasing lactose concentration. This also means that no fouling layer has formed in the course of concentration [11], since the flux at the end of one sub-trial corresponds to the one at the beginning of the following sub-trial. So it can be concluded that the driving forces that determine the flux curve are mainly the increasing osmotic pressure and the concentration polarization effects. The fact that the flux decreases to a similar extent for all five membranes also is an indication that the increasing osmotic pressure and concentration polarization due to increasing lactose concentration are primarily responsible. Towards the end of the concentration, when a lactose concentration of approximately 170 g/L has been reached, the flux is reduced by around 80% compared to the initial value at 25 g/L, with only very small differences of around 79% for the SR3D and a maximum of 83% for the NFG.

Furthermore, the retention curve of the NFG membrane indicates that the higher lactose concentration on the membrane surface due to concentration polarization leads to an improved retention. Since the concentration ratios build up by concentration polarization are reset at the beginning of each sub-trial, and thus a higher part of the lactose permeates at the beginning of each trial, the NFG does not show a consistently stable retention throughout the three sub-trials. In contrast, the other membranes provide a constantly high lactose rejection of ≥93%, which tends to improve by an average of 2% with increasing degree of concentration.

As an intermediate conclusion, it can be stated that the NFG is not suitable due to the poor lactose retention. The other four membranes all have similarly high lactose retentions, so that the decision which membranes to be used for the scale-up was based on the permeate fluxes. Since the TS40 had the lowest flows, it was not considered any further. The SR3D would be a very interesting candidate, but KOCH Membrane Systems currently does not offer 1812” spiral wound membranes, so that tests on a semi-pilot scale were not feasible and this circumstance points out another challenge researcher are faced with when membrane screening trials are planned. In conclusion, the scale-up was performed with the NFS and XN45, also because these two membranes differ in their MWCO which could have a varying outcome during the concentration of whey permeate.

### 3.3. Scale-Up and Performance Using Industrial Feeds

Two issues were the focus of the scale-up: First, comparison and evaluation of the results gained with the flat-sheet screening module with those of an 1812” spiral wound module. Second, it should be evaluated to what extent the data obtained using the lactose model system is in agreement with the data when whey permeate is concentrated, because the whey permeate contains a large number of different components in addition to lactose as stated earlier, which influence the three above mentioned thermodynamic driving forces. Moreover, since in literature it is also described that NF membranes partially retain ions [11,12,14,15], this point was also evaluated using ion quantification and conductivity measurements.

#### 3.3.1. Comparison of Module Performance

**Figure 5:**
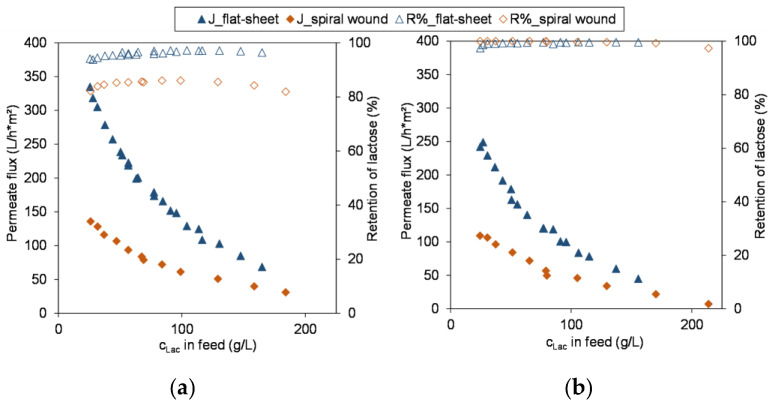
Comparison of membrane performance (flux (J) and retention (R%) of lactose (Lac)) of flat-sheet membrane versus 1812” spiral wound module for concentration of lactose model solution; (**a**) XN45 (45 °C & 3 MPa); (**b**) NFS (45 °C & 2 MPa). Operation parameters summarized in Table 3.

In Figure 5, the results of the concentration of the lactose model system using the flat-sheet membrane are compared with those using the spiral wound module. As expected for both membranes, the permeate flow rate through the spiral wound membrane is lower than through the flat-sheet membrane. Räsänen et al. [12] also compared four commercial NF membrane performances with flat-sheet trials and spiral wound modules and made comparable observations. The reason might be due to the different prevailing cross-flow conditions in the respective modules, different thickness of boundary layers, as the spiral wound module might offer a bigger resistance to the feed flow due to its multi-layered structure and the integrated spacers amplifying the effects of concentration polarization. However, van Gauwbergen et al. stated that geometry of the channel has little impact compared to the dominating effects of the spacer on the hydrodynamics [37]. It was shown that existing dead zones in spiral wound modules enhance local concentration polarization and result in a reduction of the overall membrane performance [37]. When the percentage drop in permeate flow over the course of the concentration at similar lactose concentrations is compared, the NFS shows a reduction of approximately 82% which is in very good agreement with that in the flat-sheet tests. The rejection values correspond equally well. In contrast, the use of the spiral wound module of the XN45 resulted in an approximately 11% reduced retention.

These results underline the need for scale-up experiments with drastically increased membrane areas, use of spacer and thus different hydrodynamics and/or modules closed to the ones used in industry, since filtration experiments with flat-sheet membranes have some potential weaknesses [12,38,39] as described in more detail below. Although [12] observed comparable retentions for flat-sheet as well as spiral wound modules, Schipolowski et al. [38] discussed and summarized critically the shortcomings of flat-sheet trials that are mainly due to a small effective membrane area and differences in test and flow conditions. Additionally, a certain heterogeneity in the membrane material and active layer as a result of the production process of the membrane cannot be avoided so that membrane manufacturers usually state the permeability of the membrane within a range of ±15–20% around the nominal value [20,38]. This fact might be one of the reasons why for NF membranes ranges for the MWCO are given, because the molecules and compounds that are to be separated with NF membranes are relatively small and therefore smaller fluctuations in the membrane structure might already have an influence on the membrane performance in terms of permeate flow and retention. When the available filtration area of flat-sheet membranes is limited, differences within the membrane structure can have a higher impact on the results, compared to spiral wound modules where the heterogeneity of the membrane structure is averaged over a larger area. However, Schipolowski et al. stated, that an increase in the filtration area through larger test cells does not necessarily alter the reliability since the production conditions for a nearby membrane area tends to produce a rather homogeneous membrane structure [38]. In the test setup applied in this study, the spiral wound module has an area that is almost 38 times larger than the tested flat-sheets. Taking into account the stated MWCO of the XN45 with 300–500 Da, the determined retentions of the 1812” spiral wound module seem quite plausible and suggest that the flat-sheet membrane batch covered presumably the lower MWCO range and therefore gave a higher lactose retention. As seen in the parameter studies for the NFG as an example for a looser membrane and in accordance with the literature [31,32], temperature can have a distinct effect on retention. Although this was not seen in the parameter studies for the XN45 may be due to the tighter membrane structure of the flat-sheet batch, it was seen with the spiral wound module. Therefore, for the XN45 the concentration using the spiral wound module was repeated at 20 °C. As a result, the retention was improved by almost 9%, so that these adapted operating parameters were used for the subsequent concentration of the whey permeate. As already discussed, this led to a reduced permeate flow. At both temperatures, 20 °C and 45 °C, the flux decline with the XN45 was less pronounced during the concentration than with the NFS. Precisely at 45 °C by about 71% and at 20 °C by approximately 73% although the reduction of temperature went along with an improvement in retention. It is assumed that this could be a result of the slight lactose permeation that might affect the concentration polarization layer on the membrane surface, although this was not observed with the NFG in the parameter studies, in which even more lactose passed into the permeate during the course of the concentration. On the other hand, the initial permeate flux curve in the course of concentration for the NFG was relatively steep, which makes comparability difficult.

Overall, after comparing the membrane performance of flat-sheet membranes and spiral wound membranes, it can be stated that flat-sheet membrane tests are well suited for preliminary investigations and a pre-selection, and in particular for a relative comparison of different membranes within the same test setup. Especially because in literature there are only scattered results with one [3,11,15] or up to four membranes [12]. However, it is usually difficult or impossible to compare the self-acquired data with those in the literature, since the experimental setup shows distinct differences for instance due to different test cells, plant construction and operating parameters. Therefore it was the aim of this study to give a relative comparison of ten membranes using the same test conditions. To finally be able to give reliable statements about the membrane performance, it is necessary to carry out tests with a larger filtration area, i.e., with spiral wound modules, since the production-related heterogeneity of the polymers will be statistically balanced with a larger filtration area. In particular, if the differences between the membrane performances of the flat-sheet membranes are only small, an adjustment using spiral wound modules is recommended. The “gold standard” would be using those modules and sizes that will be applied in the later industrial process, but as this is often not feasible, it is a good compromise for an approximation to use smaller module sizes where at least the flow conditions and retention might be comparable [38].

#### 3.3.2. Concentration of Industrial Whey Permeate

Another objective of this study was to verify to which extent the selected approach, initially carrying out the membrane screening with a lactose model system instead of whey permeate, is advisable and appropriate and where there might be potential limitations.

**Figure 6:**
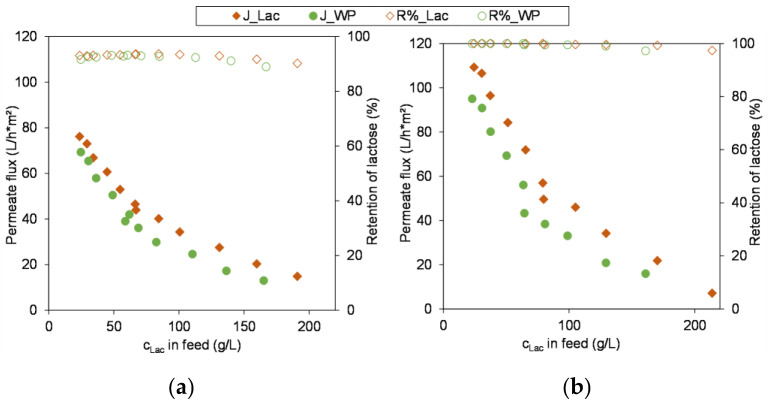
Resulting permeate flux (J) and retention (R%) of lactose during concentration of lactose model solution (Lac) versus whey permeate (WP); (**a**) XN45 (20 °C & 3 MPa); (**b**) NFS (45 °C & 2 MPa). Operation parameters summarized in Table 3.

In Figure 6 the permeate flux and the retention in the course of concentration of whey permeate are compared to that of the lactose model solution. For both membranes the lactose rejection is in very good agreement with the model system. The basic course of the permeate flux curves also agree well with the model system. Furthermore, the curves progress evenly, regardless of the subdivision into two sub-trials. This indicates that the remained proteins and peptides, which are still present in small amounts in the whey permeate (Table 1) because they cannot be retained by the 10 kDa UF membrane, do not build up a pronounced gel or fouling layer that would lead to a reduction in the flux as this was observed by [15] during the NF of sweet whey. However, in a long term process the formation of a gel layer might occur due a slowly but continuous accumulation of proteins and peptides which impairs the membrane performance. Therefore, it is recommended to conduct experiments for a much longer period of time with industrial feed in order to evaluate the long-term performance of the membrane and the process.

The fact that the permeate flux curves of the whey permeate and the lactose model system are not congruent is probably due to the high ion content of the whey permeate that affect the osmotic pressure of the feed. Timkin and Lazarev [40] have experimentally determined the osmotic pressure of a milk permeate and a lactose solution. Although the amount of lactose (4.5%) was much higher than the amount of ions (0.62%) the osmotic pressure of the milk permeate was approximately double as high as of the lactose solution indicating a pronounced effect of the milk ions on the osmotic pressure [40]. Since the osmotic pressure of the feed reduces the driving force of the process, this leads to a reduced permeate flow.

**Figure 7:**
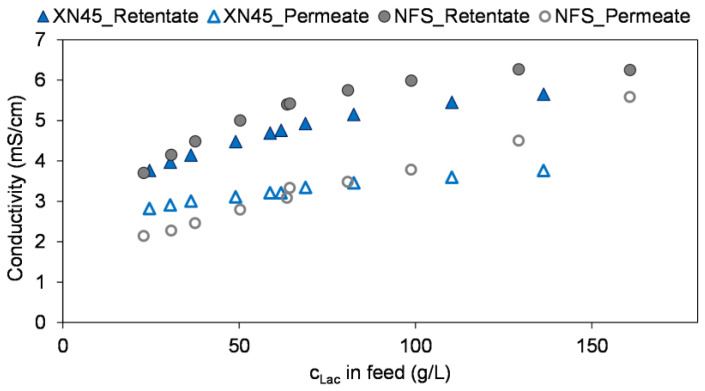
Comparative comparison of the course of conductivity in retentate and permeate during the concentration of lactose in whey permeate with XN45 and NFS membrane.

As shown in Figure 7, the conductivity increases in the course of concentration both in the retentate and in the permeate, with the conductivity in the retentate being significantly higher than that of the permeate right from the start. This means that in addition to the lactose, some of the ions are also retained and concentrated which was also confirmed by ion chromatography performed.

**Figure 8:**
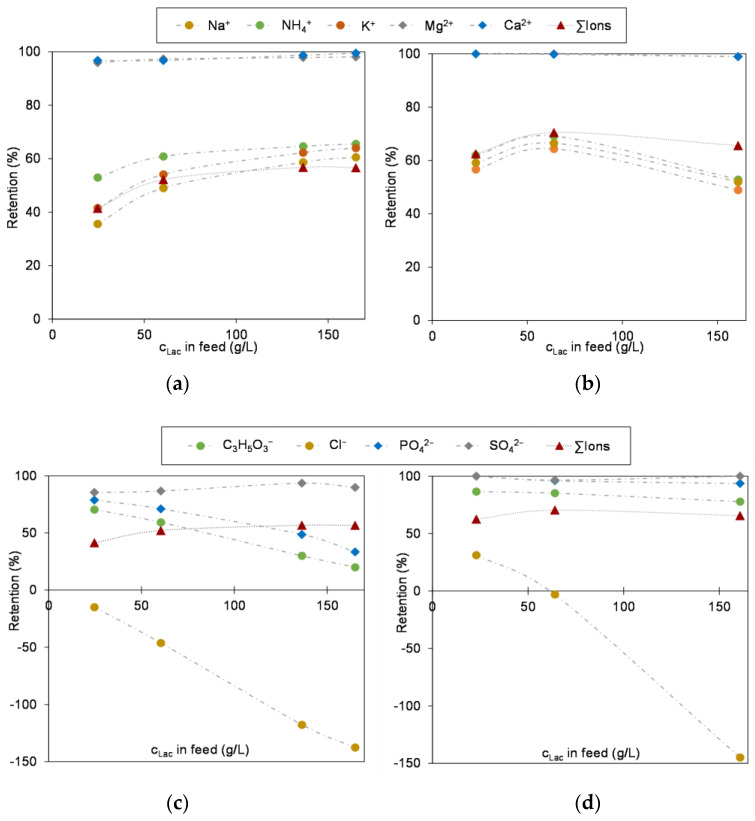
Retention of ions during concentration of lactose in whey permeate; (**a**) cations, membrane: XN45; (**b**) cations, membrane: NFS; (**c**) anions, membrane: XN45; (**d**) anions, membrane: NFS. Operation parameters summarized in Table 3.

In Figure 8 the retentions of the analyzed ions are visualized. In accordance with literature, the divalent ions are almost completely (XN45) or completely (NFS) retained, while monovalent ions partially pass through the membrane. A special case are the chloride ions that show a negative retention due to the well-known Donnan effect. As the divalent anions cannot pass the membrane and thus the electrochemical potential increases on the feed side during the concentration, the monovalent chloride ions, as they are able to pass the membrane, are especially forced to permeate in order to equalize this state aiming towards an electro neutrality, even against its concentration gradient [11,14,15]. Finally, the observed chloride concentration in the permeate is higher than in the feed. Additionally, the increase in conductivity of the permeate and the course of retentions of ions demonstrate that increasing salt concentrations lead to a decrease in the retention of the ions due to electrostatic interactions [14,15,26]. Both in Figure 7 and Figure 8 can be seen that the ion retention is less pronounced with the XN45 and thus a higher percentage of ions passes through the membrane. This results in a stronger partial demineralization in the course of lactose concentration compared to the NFS.

Precisely, looking at the removal of single ions in dependency of the used membrane the following picture is drawn—lactic acid: 51% with the XN45 versus 20% with the NFS; chloride: 93% versus 70%; sodium: 42% versus 26% and potassium: 38% versus 24%. In turn this could explain why the permeate flux curves of the lactose and whey permeate feeds using the XN45 are in better agreement than the curves of the NFS. Since the soluble ingredients, especially lactose and ions, determine the osmotic pressure of the feed [19], the deviations between the curves increase in the course of concentration. Overall, it can be concluded that the course of the flux as the lactose is concentrated is basically a function of the concentration polarization effects, which in turn are determined by the osmotic pressure [11,35,40]. Thus, the lactose model solution can give a good approximation of the general membrane performance in terms of permeate flow and retention. However, since the raw material milk and its specific ionic composition are subject to a certain range of fluctuation as discussed earlier, trials with industrial whey feeds are necessary for a final evaluation and set-up of the whole process.

**Figure 9:**
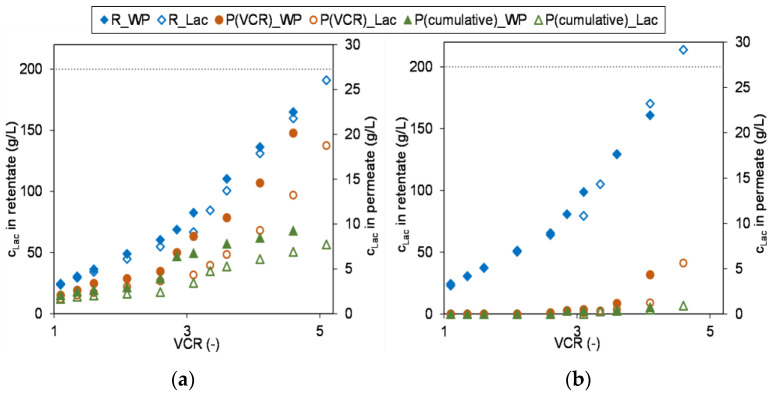
Evolution of lactose concentration in retentate (R), permeate (P(VCR)) and cumulative permeate (P(cumulative)) during the concentration of lactose model system (Lac) and whey permeate (WP); (**a**) membrane: XN45; (**b**) membrane: NFS.

Another factor that might be relevant for the design of the process is the composition of the resulting permeate. Especially if for instance legislative specifications regarding the waste water treatment have to be obeyed or if the permeate is to be used as rinsing water or for DF in order to economize resources. Figure 9 shows the increase in lactose concentration in the retentate (y-primary axis) and permeate (y-secondary axis) with raising VCR for both feeds. Additionally, the figure visualizes the lactose concentration ratios, i.e., how they prevail directly at the membrane (permeate at the specific VCR) and how it emerges within the total cumulative permeate (P_cumulative). With the XN45, a small portion of the lactose permeates continuously, whereby this increases noticeably with progressive lactose concentration in the retentate, but is has a less pronounced influence on the cumulative permeate. When lactose of the model solution was concentrated to approximately 160 g/L, around 6.2% of lactose passed into the permeate. For whey permeate as feed, the ions appear to increase the lactose loss due to their concentration polarization enhancing effect, which is then about 10.4%. In contrast, the NFS exhibits complete retention over a longer period of time, up to around 80 g/L lactose within the feed. With rising concentrations, only minimal permeation of lactose takes place, so that for both feeds, the lactose model system (0.3%) and the whey permeate (0.7%), less than 1% lactose passes through the membrane and thus the resulting permeate is almost lactose-free and could e.g., be used for pre-cleaning, DF or other processes within the dairy.

## 4. Conclusions

The issue of identifying suitable membranes for an ecologically and economically efficient process of lactose concentration from whey permeate was realized in three stages including scale-up. First, 10 polymer membranes as flat-sheets were examined for their suitability within a TMP-screening, whereas a high to complete lactose retention in combination with a high flux were defined as the decision criterion. These trials were carried out using a lactose model solution, as whey permeate composition can vary widely, which could affect the results. Minimal coherence was found between the MWCO specified by the manufacturers and the maximum achievable flux underlining the necessity of preliminary trials. The retention of lactose was basically in agreement with the expectation based on the MWCO. In a second step detailed parameter studies were carried out with five promising selected membranes in order to investigate the influence and magnitude of the process variables temperature, TMP and increasing lactose content. Both a TMP increase from 2.0 to 3.0 MPa and a temperature increase from 20 °C to 45 °C led to significantly higher permeate fluxes, with the temperature influence being more pronounced and the combination of both operating parameters achieving the strongest effects. However, some membranes are limited in terms of their maximum tolerable process conditions, so that the synergistic effects of temperature and TMP cannot be used for them. With increasing lactose concentrations in the course of the concentration, there was a steady decrease in flux due to the increase in osmotic pressure and concentration polarization. In the last scale-up stage the plausibility and reproducibility of the results gained with the flat-sheet membranes were verified with spiral wound modules since flat-sheet results might be limited due to the small filtration area and diverse flow conditions compared to spiral wound modules used in industry. In addition, trials were carried out with real, industrially produced whey permeate. It was shown that the permeate flux values with the spiral wound modules were generally lower than with the flat sheets. With the NFS, the lactose retention was in good agreement with the preliminary tests. For the XN45, the retention was lowered with the spiral wound module at 45 °C, whereas a temperature reduction to 20 °C was accompanied by improved retention. The comparison of the membrane performance for the different feeds revealed that the lactose model solution is very well suited for preliminary studies in order to reproducibly depict the basic behavior of whey permeate. With increasing volume concentration ratio, there are more pronounced deviations between the flux curves of the model system and the whey permeate, because ions are also partially retained, which contribute to the amplification of the concentration polarization phenomena and the osmotic pressure of the concentrate. The more ions were retained, the more pronounced the differences were. The choice of membranes should be made depending on the objective which means whether the focus is more on complete lactose retention or partial demineralization. It could be shown that both membranes are basically well suited for concentration of lactose up to about 200 g/L. With the NFS, lactose can be concentrated with a loss of less than 1%, so that the resulting permeate is almost lactose-free and can be used for instance for upstream diafiltration steps or for cleaning. In contrast, a higher degree of demineralization can be achieved with the XN45, which is associated with higher lactose losses. Finally, in order to implement an ecologically and economically efficient process it is recommended to carry out trials with (a) industrial whey permeate and (b) performing long-time experiments, whereby the here presented data might provide a good starting point.

## Figures and Tables

**Table 1:** Average composition of the sweet whey permeate used.

	Sweet Whey Permeate
pH	6.5
conductivity (mS/cm)	3.7
Dry matter (%)	3.2
Lactose (g/L)	26.3 (→ 25 for lactose model solution)
Protein (%)	0.1
Sodium (mg/L)	169.5
Potassium (mg/L)	776.8
Ammonium (mg/L)	70.1
Magnesium (mg/L)	29.9
Calcium (mg/L)	193.0
Lactic acid (mg/L)	376.6
Chloride (mg/L)	464.5
Nitrate (mg/L)	45.6
Phosphorus (mg/L)	606.0
Sulfate (mg/L)	60.6

**Table 2:** Overview of the nanofiltration membranes used in this study.

Model	Manufacturer	MWCO ^1^ (g/mol)	Ø MWCO ^1^ (g/mol)	Membrane Chemistry ^2^	Max. Operating Temperature (°C)	Max. Operating Pressure (MPa)	
TS 50	MANN+ HUMMEL	200–300	250	Thin-Film Polypiperazine	45	4.1	
TS 40		200–300	250				
XN45		300–500	400				
NP030		500–600	550	PES TFC	50	4.0	
NFS	Synder Filtration	100–250	175	Proprietary PA TFC	50	if T < 35 °C: 4.1	if T > 35 °C: 3.0
NFX		150–300	225				
NFW		300–500	400				
NDX		500–700	600				
NFG		600–800	700				
SR3D	KOCH Membrane Systems	200	200	TFC PA	45	4.5	

^1^ MWCO = molecular weight cut-off; ^2^ PES = polyethersulfone; TFC = thin-film composite; PA = polyamide.

**Table 3:** Overview of parameter settings and membrane used for experimental setup.

Stage	Membrane Module	Filtration Mode	Tested Membranes	Feed	T (°C)	TMP ^1^ (MPa)	Initial Feed-c_(Lac)_ (g/L)
TMP-Screening	Flat-sheet	recirculation	all	lactose model solution	20	0.5→3.5→0.5	25
Parameter studies	Flat-sheet	concentration	TS40; XN45; NFS; NFG; SR3D	lactose model solution	20	2	25
						3	
					45	2	
			XN45; SR3D; TS40			3	25
			XN45; SR3D				50
							75
			NFS; NFG; TS40			2	50
							75
Scale-up	1812’’ spiral wound	concentration	NFS	lactose model solution	45	2	25–75
				whey permeate			
			XN45	lactose model solution	45	3	
				lactose model solution	20	2	
				whey permeate			

^1^ TMP = transmembrane pressure.

## Data Availability

Not applicable.
